# Neuroprotective and Neurorescue Mode of Action of *Bacopa monnieri* (L.) Wettst in 1-Methyl-4-phenyl-1,2,3,6-tetrahydropyridine-Induced Parkinson’s Disease: An *In Silico* and *In Vivo* Study

**DOI:** 10.3389/fphar.2021.616413

**Published:** 2021-03-16

**Authors:** Babita Singh, Shivani Pandey, Mohammad Rumman, Shashank Kumar, Prem Prakash Kushwaha, Rajesh Verma, Abbas Ali Mahdi

**Affiliations:** ^1^ Department of Biochemistry, KGMU, Lucknow, India; ^2^ Molecular Signaling and Drug Discovery Laboratory, Department of Biochemistry, Central University of Punjab, Punjab, India; ^3^ Department of Neurology, KGMU, Lucknow, India

**Keywords:** neurodegeneration, antioxidants, *Bacopa monnieri*, substantia nigra, iNOS

## Abstract

**Ethnopharmacological Relevance:** Parkinson’s disease (PD) is characterized by progressive death of dopaminergic neurons. The presently used medicines only tackle the symptoms of PD, but none makes a dent on the processes that underpin the disease’s development. Herbal medicines have attracted considerable attention in recent years. *Bacopa monnieri* (L.) *Wettst* (Brahmi) has been used in Indian Ayurvedic medicine to enhance memory and intelligence. Herein, we assessed the neuroprotective role of *Bacopa monnieri* (L.) *Wettst* on Parkinson’s disease.

**Aim of the Study:** *Bacopa monnieri* (L.) *Wettst*, a medicinal herb, is widely used as a brain tonic. We investigated the neuroprotective and neurorescue properties of *Bacopa monnieri* (L.) *Wettst* extract (BME) in 1-methyl-4-phenyl-1,2,3,6-tetrahydropyridine (MPTP)-induced mice model of PD.

**Materials and Methods:** The mice model of MPTP-induced PD is used in the study. In the neuroprotective (BME + MPTP) and neurorescue (MPTP + BME) experiments, the animals were administered 40 mg/kg body weight BME orally before and after MPTP administration, respectively. Effect of BME treatment was evaluated by accessing neurobehavioral parameters and levels of dopamine, glutathione, lipid peroxide, and nitrites. An *in silico* study was performed using AutoDock Tools 1.5.6 (ADT).

**Results:** A significant recovery in behavioral parameters, dopamine level, glutathione level, lipid peroxides, and nitrite level was observed in BME-treated mice. Treatment with BME before or after MPTP administration has a protective effect on dopaminergic neurons, as evidenced by a significant decrease in GFAP immunostaining and expression of inducible nitric oxide synthase (iNOS) in the substantia nigra region; however, the degree of improvement was more prominent in mice receiving BME treatment before MPTP administration. Moreover, the *in silico* study revealed that the constituents of BM, including bacosides, bacopasides, and bacosaponins, can inactivate the enzyme monoamine oxidase B, thus preventing the breakdown of MPTP to MPP+.

**Conclusion:** Our results showed that BME exerts both neuroprotective and neurorescue effects against MPTP-induced degeneration of the nigrostriatal dopaminergic neurons. Moreover, BME may slow down the disease progression and delay the onset of neurodegeneration in PD.

## Introduction

*Bacopa monnieri* (L.) *Wettst* (Brahmi, BM) is a reputed drug of Ayurveda (Bammidi et al., 2011). It belongs to *Scrophulariaceae* family, which represents 220 genera with more than 4,500 species, and typically grows in the wetlands of southern India and Australia. Of all the Indian herbs, BM was, and still considered as, the premier herb for treating brain disorders and age-related mental decline as well as for improving cognitive functions. BM has been utilized as a brain tonic, diuretic, antidepressant, revitalizer of sensory organs, cardiotonic, antianxiety, and anticonvulsant agent (Chopra et al., 1956, 2004). Animals treated chronically with BM extract showed improved acquisition skills and improved retention in learning tasks (Prisila Dulcy et al., 2012; Yadav et al., 2014a; Yadav et al., 2014b; Balaji et al., 2015).

The main constituents of BM are dammarane type of triterpenoid saponins called bacosides, with jujubogenin or pseudojujubogenin moieties as a glycone unit. The main alkaloids include brahmine, nicotine, and herpestine, along with D-mannitol, apigenin, hersaponin, monnierasides I–III, cucurbitacins, and plantainoside B. Bacosides are a family of 12 acknowledged analogs. Novel saponins called bacopasides I–XII have been identified recently. Bacoside A, which is a blend of bacoside A3, bacopaside II, bacopasaponin C, and a jujubogenin isomer of bacosaponin C, is the most studied compound of BM (Aguiar and Borowski, 2013).

BM extract as well as its isolated compounds has been studied extensively in animal models of various diseases. A previous study showed that Bacoside A could reduce oxidative stress in the brain by enhancing the activities of superoxide dismutase (SOD), catalase, glutathione peroxidase (GPx), and glutathione reductase (GSR) (Commens, 1983). Moreover, in a *Caenorhabditis elegans* model of 6-hydroxydopamine (6-OHDA)-induced Parkinson’s disease (PD), BM extract could reduce α-synuclein aggregation by inducing the expression of stress-buffer protein hsp-70 (Chowdhuri et al., 2002; Jadiya et al., 2011). Our previous study showed that chronic 1-methyl-4-phenyl-1,2,3,6-tetrahydropyridine (MPTP) administration induces PD-like symptoms in mice and cotreatment with BM extract could ameliorate the motor defects and increase the number of dopaminergic neurons in the substantia nigra of PD animals (Singh et al., 2017). We also reported that the neuroprotective effect of BM extract was mediated via upregulation of antiapoptotic protein Bcl2 (Singh et al., 2017). In a rat model of rotenone-induced PD, BM extract could ameliorate motor defects by reducing oxidative stress in the substantia nigra, hippocampus, striatum, cortex, and brain stem regions.

BM extract has low toxicity and exerts apparent beneficial effects as a nootropic (Pravina et al., 2007; Aguiar and Borowski, 2013). BM extract is used as a dietary supplement (KeenMind or CDRI08, Soho Flordis International) and is approved by the Food and Drug Administration (FDA). Although BM is widely available and BM extract is used as herbal medicine, the mechanisms of action of BM have yet to be delineated.

Parkinson’s disease (PD) is the second most common neurodegenerative disorder, and about 1% of the population over 60 years of age is affected by this disease (de Rij et al., 2000). In PD, the loss of dopamine-producing neurons is mainly responsible for PD-associated symptoms. Since the neurotransmitter dopamine is associated with the motor activity, therefore, loss of dopaminergic neurons leads to tremors, muscle rigidity, and bradykinesia. Moreover, PD also affects cognition, mental state, sleep, personality, and behavior leading to depression and anxiety (Cheng et al., 2010). The etiology of PD is still not clearly understood. At present, the available treatments for PD improve few symptoms of the disease. However, these treatment modalities have suboptimal efficacy and low efficiency. Thus, there is an urgent need to develop novel neuroprotective or disease-modifying treatments for PD. Natural products or herbal compounds are of particular interest as they can be used to develop novel drugs that could help in preventing or delaying the PD-associated neurodegeneration.

The present study aimed to evaluate the effects of pre- and posttreatment with BM extract on PD-associated motor defects and neuroinflammation employing an MPTP-induced mice model of PD. Further, the study also aimed to explore the neuroprotective mode of action of BM extract through an *in silico* study. Our results suggest that both pretreatment (neuroprotective) and posttreatment (neurorescue) with BM extract ameliorate the motor defects in PD mice. Moreover, both pre- and posttreatment with BM extract reduce oxidative stress, increase the dopamine levels, decrease the inflammation, and suppress microglial activation in the substantia nigra region of the MPTP-treated mice brain. Employing an *in silico* approach, we identified the probable active BM phytoconstituents that might be involved in its neuroprotective and neurorescue properties.

## Materials and Methods

### Animals and Treatment

In this study, Swiss albino mice were used. Animals were obtained from the breeding colony of Indian Institute of Toxicology Research (IITR), Lucknow, India. All animal experiments were performed after obtaining approval from the Institutional Animal Ethics Committee (IAEC, letter no. 39/IAH/PHARMA/14). Animals were maintained under 12–12 h light-dark cycles with an ambient temperature (25°C) and controlled humidity, along with free access to drinking water and pellet diet (Hindustan Lever Ltd., Mumbai, India).

### Experimental Schedule

*Bacopa monnieri* (L.) *Wettst* extract (BME) was purchased from Natural Remedies Pvt. Ltd., Bangalore, India (BM/10015).

For experimental design, mice were randomly divided into the following four groups (n = 8 in each group): Group I: animals received 0.5 ml of normal saline (intraperitoneal (i.p.) for 3 weeks) served as control or vehicle group; Group II: animals received MPTP (30 mg/kg body weight, i.p.) on the 8^th^ day of the experiment, twice with a gap of 16 h to induce PD-like symptoms (MPTP group) (Yadav et al., 2014a; Yadav et al., 2014b); Group III: animals received BME treatment (40 mg/kg body weight/day, p. o.) for 1 week (Singh et al., 2016; Rai et al., 2003; Singh et al., 2017; Singh et al., 2020) followed by MPTP treatment on the 8^th^ day of the experiment, twice with a gap of 16 h, and BME treatment continued for another 2 weeks (neuroprotective experiment, BME + MPTP group); and Group IV: animals received MPTP on the first day of the experiment followed by BME treatment (40 mg/kg body weight/day, p.o.) for 3 weeks (neurorescue experiment, MPTP + BME group).

At the end of the entire treatment, neurobehavioral tests were performed to evaluate the motor functions. After that, the animals were sacrificed and the brain was dissected out for further biochemical analysis.

### Neurobehavioral Studies

#### Footprinting Test

Footprint analysis was performed to assess limb coordination in all mice groups as described previously (Yadav et al., 2014a; Yadav et al., 2014b). Briefly, animals were trained to walk across a white sheet of paper without stopping. At end of the drug treatment, the forepaw of mice was dipped in blank ink and the animals were allowed to walk freely on a white sheet of paper. The stride length was measured as the distance between the centers of the ipsilateral adjacent footprints (Yadav et al., 2014a; Yadav et al., 2014b).

#### Rotarod Test

The rotarod test was carried out using the Rotomex (Columbus Instruments, USA) instrument to evaluate the motor coordination in animals. The apparatus consisted of an iron rod with nonslip surface (3 cm diameter, 30 cm length). Prior to the drug treatment, animals were trained by placing them on the rotating rod (10 rpm) for 300 s. After the treatment, the fall time of animals from the rotating rod was recorded (Rai et al., 2016).

#### Grip Strength Test

To measure the forelimb strength in mice, the grip strength test was used as described previously (Terry et al., 2003).

### Neurochemical Studies

After the behavioral tests, mice were anesthetized using ketamine/xylene (60 mg/kg) and sacrificed via cervical dislocation followed by decapitation to ensure minimum pain. The whole brain was dissected out and frozen directly until further analyses. The substantia nigra was carefully dissected out from both the hemispheres of each mice brain and stored at −80°C until further use.

Oxidative stress was evaluated by measuring the levels of malondialdehyde (MDA), nitrite, and glutathione (GSH) in the substantia nigra region.

#### Malondialdehyde (MDA) Estimation

Malondialdehyde levels can be the indicator of lipid peroxidation. MDA is a reactive three-carbon di-aldehyde formed as a byproduct of polyunsaturated fatty acid (PUFA) peroxidation. In the brain tissue, the lipid peroxidation level (LPO) was estimated according to the method described previously (Ohkawa et al., 1979) with few modifications. Briefly, the assay mixture was incubated at room temperature for 5 min followed by the addition of 20% acetic acid (0.6 ml) and further incubation for another 5 min. After this, 0.8% thiobarbituric acid (TBA) (0.6 ml) was added to the mixture and incubated in a boiling water bath for 1 h. The reaction mixture was cooled and centrifuged, and the absorbance was recorded at 532 nm. LPO was measured as nanomoles MDA/mg protein.

#### Nitrite Estimation

Nitrite levels were determined in the supernatant of the tissue homogenate as described previously (Granger et al., 1996). Briefly, tissue homogenate was incubated with sodium nitrite (10 mM), ammonium chloride (0.7 mM), and Griess reagent (0.1% N-naphthylethylenediamine and 1% sulfanilamide in 2.5% phosphoric acid). The reaction mixture was incubated at room temperature for 30 min, and the absorbance was measured at 540 nm. The nitrite content was calculated using a standard curve of sodium nitrite (10–100 μM). Nitrite levels were expressed as µmoles/ml.

#### Glutathione (GSH) Estimation

GSH was estimated in the tissue homogenate by 5,5′dithiobis-(2-nitrobenzoic acid) (DTNB)-glutathione reductase coupled assay as described previously (Anderson 1985). Briefly, 1.0 ml of the brain tissue homogenate was deprotonated by adding 1.0 ml of 10% TCA and centrifuged at 6000 g for 5 min. 0.5 ml aliquot from the clear supernatant was mixed with 0.5 ml double distilled water. Thereafter, 2 ml of 0.4 M Tris buffer and 0.1 ml DTNB were added to it with continuous mixing. GSH reacts with DTNB to produce a yellow-colored chromogen. The absorbance was measured at 412 nm. GSH content in the sample was calculated using a standard curve (GSH 200–1,600 nmole), and the results were expressed as nmole/g tissue.

#### Dopamine Estimation by HPLC

The dopamine (DA) level was estimated in the isolated substantia nigra homogenate using HPLC (Yadav et al., 2014a; Yadav et al., 2014b). Briefly, the samples were homogenized in 0.17 M perchloric acid using a Polytron homogenizer. The homogenates were centrifuged at 33000 g (Biofuge Stratos, Heaureas, Germany) at 4°C.Then, 20 µL of the supernatant was injected into an HPLC pump (Model 1,525, binary gradient pump) fitted with a C18 column (Spherisorb, RP C18, 5 mm particle size, 4.6 mm i.d. × 250 mm at 30°C) connected to an ECD (Model 2,465, Waters, Milford, MA, USA) at a potential of +0.8 V with a glassy carbon working electrode vs. Ag/AgCl reference electrode. The mobile phase consisted of 32 mM citric acid, 12.5 mM disodium hydrogen orthophosphate, 1.4 mM sodium octyl sulfonate, 0.05 mM EDTA, and 16% (v/v) methanol (pH 4.2) at a flow rate of 1.2 ml/min. The chromatogram was recorded and analyzed using Empower software (Version 2.0).

### Enzyme-Linked Immunosorbent Assay (ELISA)

TNF-α protein levels were measured in the substantia nigra homogenates using a commercially available kit for ELISA (Diaclone).

### GFAP Immunostaining

Sections of the substantia nigra region were blocked in blocking buffer (PBS containing 2% normal goat serum) for 2 h. Sections were then incubated with monoclonal mouse anti-GFAP antibody (1:1,000) overnight at room temperature. Then, sections were washed with PBS thrice and incubated with biotinylated anti-mouse IgG (1:500) for 2 h at room temperature and subsequently in avidin peroxidase (1:500 dilution) for 2 h. DAB (Sigma) was used to visualize the immunoreactivity. The stained sections were dehydrated and mounted using a coverslip. For quantification, images were acquired on the Nikon Eclipse TiBR imaging system using a ×10 objective. ImageJ software was used to determine the fraction of GFAP positive area.

### Gene Expression Analysis by Quantitative Real-Time PCR

Total RNA was isolated from the substantia nigra region using TRIzol reagent. Genomic DNA was removed using RNase-free DNase (Ambion). RNA pellets were resuspended in DEPC-treated water (Ambion). Equal amounts of RNA were reverse transcribed using the Superscript first-strand cDNA synthesis kit with Oligo-dT (Invitrogen, USA) and diluted in nuclease-free water (Ambion) to a final concentration of 10 ng/μL. Real-time q-PCR was performed to detect changes in mRNA expression using the SYBR green and ABI Prism 7900 HT Sequence Detection System (Applied Biosystems; Foster City, CA). Beta-actin was used as internal control. Relative expression was calculated using the delta Ct method (Tiwari et al., 2014).

The primers used for qPCR were iNOS forward 5′-CCCTTCCGAAGTTTCTGGCAGCAGC-3′and iNOS reverse 5′-GGC​TGT​CAG​AGC​CTC​GTG​GCT​TTG​G-3’.

### *In Silico* Study

#### Ligand Screening and Preparation

NCBI PubChem compounds database and literature on BM phytochemicals were used to take an idea for the selection of active phytoconstituents from BM for the present study (http://www.ncbi.nlm.nih.gov/pccompound). As per the literature, bacosides, bacopasides, and bacopasaponins form the major proportion of active phytochemicals in BM. The PubChem compound search tool was used to find the natural and/or synthetic analogs of the active phytoconstituents. Thirty-one analogs of bacosides (N = 2), bacopasides (N = 20), and bacopasaponins (N = 9) were used for further *in silico* study. The standard known inhibitors for different targeted proteins were retrieved from the literature. The 3D/2D structure of phytochemicals and various inhibitors of their respective protein were retrieved from NCBI PubChem in SDF format. Open Babel molecule format converter was used to perform conversion of 2D structure into 3D conformation (O’Boyle et al., 2011)

#### Screening and Preparation of Ligand Receptor

The 3D structures of various ligand-receptor proteins were downloaded from RCSB-protein data bank in PDB file (Berman et al., 2000). The bulkier structure of MAO-A protein (enzyme) was divided into two (Chain A and Chain B) separate PDB files. Chain C (Nrf2) of PDB 3ZGC and Chain A (neuronal calcium sensor 1) of PDB 5AER were deleted to obtain KEAP1 and D2 dopamine receptor proteins, respectively. Protein models were cleaned and optimized by removing ligands as well as other heteroatoms (acetate ion and H_2_Ofor KEAP1; Mg, S-adenosylmethionine, 3,5-Dinitrocatechol, K and H_2_O for COMT; N-[3-(2,4-Dichlorophenoxy)Propyl]-N-Methyl-N-Prop-2-Ynylamine and FAD for MAO-A; FAD, H_2_O and (5R)-5-{4-[2-(5-ethylpyridin-2-yl)ethoxy]benzyl}-1,3-thiazolidine-2,4-dione for MAO-B; 5′-S-(3-{[(3R)-1,2,3,4-tetrahydroisoquinolin-3-ylcarbonyl]amino}propyl)-5′-thioadenosine, 2-Amino-2-Hydroxymethyl-Propane-1,3-Diol and H_2_O for PNMT; L-Dopamine, Adenosine-3′-5′-diphosphate and H_2_O for PST; β-D-Galactose, SO_4_, Uridine-5′-Diphosphate, Mn and H_2_O for UGT; 1-benzyl-1H-indole-2,3-dione, Na, 1,2-Ethanediol, Guanidine and H_2_O for ALDH1A1; 3-chloro-5-ethyl-N-{[(2S)-1-ethylpyrrolidin-2-yl]methyl}-6-hydroxy-2-methoxybenzamide and Maltose for D3 dopamine receptor and Ca, K and H_2_O for D2 dopamine receptor). Energy minimization was done by using the Swiss-PDB Viewer (v4.1) software.

#### Molecular Docking Stimulation

For docking experiments, the ligand-receptor proteins and the ligands were loaded into AutoDock Tools 1.5.6 (ADT) (Sanner 1999). Gasteiger partial charges assigned after merging nonpolar hydrogen and torsions were applied to the ligands by rotating all rotatable bonds. Docking calculations were carried out on the protein models. Polar hydrogen atoms, Kollman charges, and solvation parameters were added with the aid of AutoDock tools. AutoDock 4.2 offers the option of three search algorithms to explore the space of active binding with different efficacy. We used the Lamarckian genetic algorithm (LGA) in this study.

#### Visualization of the Results

LigPlot^+^ was used to visualize the hydrogen bonds as well as the exact distance between residues of KEAP1 (Kelch-like-ECH-associated protein 1) receptor protein and different atoms of bacopaside-XII. PyMOL (v1.1) software was used for visualization of the interaction pattern in different receptor protein and ligand. Molecular surface structure was obtained by PyMOL software.

#### Statistical Analysis

Data are represented as mean ± standard deviation (SD). Comparison between the treated and the untreated groups was performed using one-way analysis of variance (ANOVA), followed by the Student–Newman–Keuls test (InStat3 package program). *p* < 0.05 was considered statistically significant.

## Results

### Neurobehavioral Studies

**FIGURE 1:**
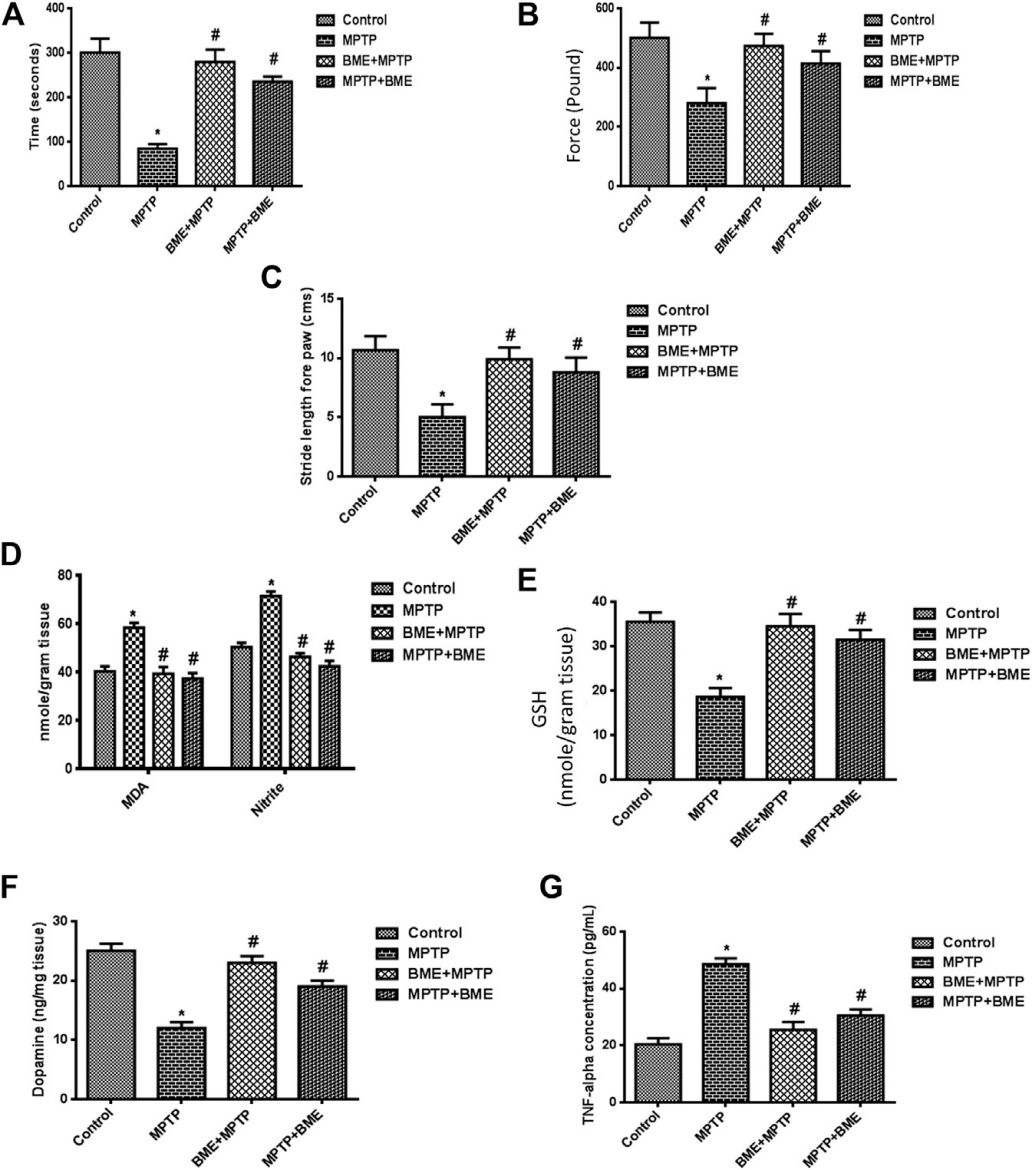
Assessment of neurobehavioral and biochemical parameters in treated and untreated groups. **(A)** The rotarod test was performed in all the groups of animals, and a significant perfection in the time of stay on the rotarod was found in BME pretreated mice compared with the MPTP group. **(B)** The grip strength test was significantly reduced in the MPTP mouse as compared with control. The protective effect was found on forelimb grip strength in pre- and posttreatment of the BME group (BME + MPTP and MPTP + BME). **(C)** Effect of *Bacopa monnieri* (L.) *Wettst* extract (BME) on stride forepaw length. MPTP mice showed decreased stride forepaw length as compared with control. **(D)** Levels of lipid peroxides and nitrite. **(E)** Effect of BME treatment (before and after) in mice on the GSH level. **(F)** Levels of dopamine. **(G)** Effect of pre- and posttreatment of BME extract on the TNF-α level. Values are expressed in mean ± SD. *: compared with the control group, #: compared with the MPTP-treated group. *p* < 0.05 was considered as significant.

To evaluate the efficacy of BME in ameliorating MPTP-induced behavioral deficits, we studied neurobehavioral changes using rotarod test, grip strength test, and foot printing test. MPTP-induced mice exhibited a significant decrease (*p* < 0.05) in the time spent on the rotarod as compared with control mice (Figure 1A), which is in agreement with previous studies (Singh et al., 2020). Both, pre- and posttreatment with BME significantly increased the time spent on the rotarod in MPTP-treated mice. Interestingly, mice receiving BME treatment before MPTP lesioning (BME + MPTP group) exhibited a more significant (*p* < 0.001) increase compared with mice receiving BME treatment after MPTP lesioning (MPTP + BME) (*p* < 0.05). Further, grip strength was significantly decreased in mice treated with MPTP compared with the control group (Figure 1B). An improvement in the grip strength was observed in both pretreatment (BME + MPTP) and posttreatment (MPTP + BME) groups compared with the MPTP-induced group. Similar to the rotarod test, the increase in the pretreatment group (BME + MPTP) was significantly more compared with the posttreatment group (MPTP + BME) in the grip strength test. In the footprinting test, we observed that MPTP treatment induced walking errors in mice (*p* < 0.05). Pre- and posttreatment with BME ameliorated the walking errors in MPTP-induced mice (Figure 1C). Taken together, these results suggest that both pre- and posttreatment with BME could ameliorate the motor deficits in MPTP-treated PD mice.

### Biochemical Analysis

In the MPTP-induced group, a significant increase (*p* < 0.001) in the levels of lipid peroxides and nitrite in the substantia nigra region was observed compared with control mice. Both, pre- and posttreatment with BME decreased the levels of lipid peroxides and nitrite; however, the decrease was more significant in the BME + MPTP group (*p* < 0.001) than in the MPTP + BME group (*p* < 0.05) (Figure 1D). Moreover, a significant decrease (*p* < 0.001) in the GSH level (Figure 1E) was observed in the MPTP-induced group compared with the control group. Pre- or posttreatment with BME increased the GSH levels in MPTP-treated mice. The increase in the BME + MPTP group was more significant (*p* < 0.001) than the MPTP + BMP group (*p* < 0.05). As shown in Figure 1F, a significant decrease in the DA level was observed in MPTP-induced mice (*p* < 0.001) when compared with the control group, indicating death of dopaminergic neurons in the MPTP-induced animals. Pre- and posttreatment with BME restored the levels of DA. The effect was more pronounced in the BME + MPTP group (*p* < 0.01) than in the MPTP + BME group (*p* < 0.05). A significant increase (*p* < 0.001) in the TNF-α level was observed in the substantia nigra of the MPTP-induced group as compared with the control group, which was restored significantly in both pre- and posttreated groups (*p* < 0.01 and *p* < 0.05, respectively) (Figure 1G).

### Immunohistochemistry

**FIGURE 2:**
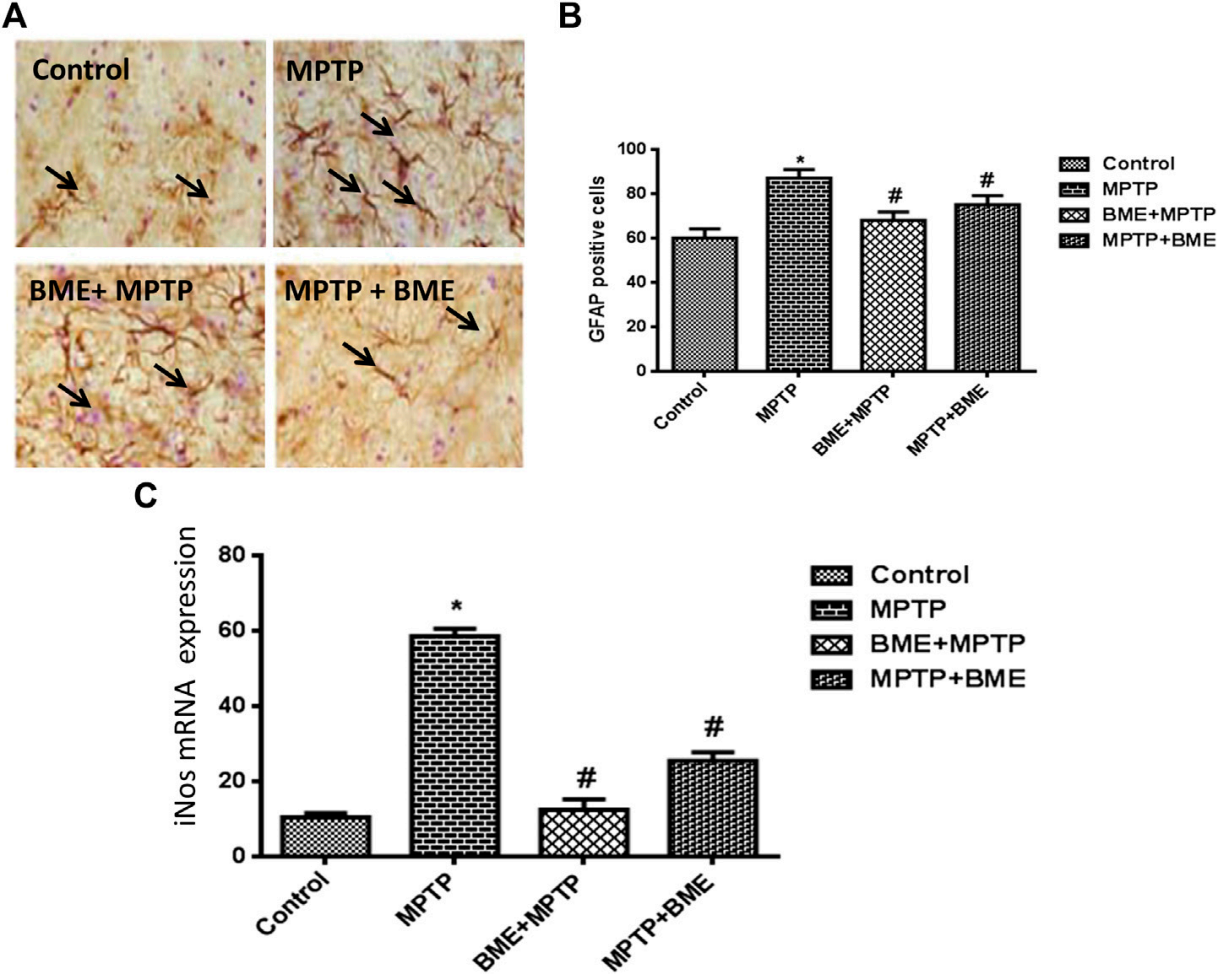
Immunohistochemical studies of astrocytes and quantitative estimation of iNOS mRNA. **(A)** Immunostained representative in brain tissue sections of the substantia nigra (SN), showing astrocyte marker GFAP immunoreactivity in control and all treated animals. **(B)** Bar diagram showing the number of GFAP fibers in the SN region of different groups. Each bar represents mean ± SD. Data are shown as percentages relative to untreated controls. **(C)** q-PCR estimation of iNOS mRNA, beta-actin was used as internal normalizer. *p* < 0.05 was considered as significant. *: comparison of the control group and # comparison of the MPTP-treated group. *p* < 0.05 was considered as significant.

The neuroprotective/neurorescue effect of BME was assessed by staining the activated astrocytes. In MPTP-induced mice, the number of GFAP positive astrocytes was significantly higher compared with the control animals (Figures 2A,B). In both MPTP + BME and BME + MPTP groups, a significant decrease in GFAP immunoreactivity was observed as compared with the MPTP group (Figures 2A,B). The decrease in GFAP positive astrocytes in SNpc of BME + MPTP and MPTP + BME group confirms the neuroprotective/neurorescue action of BME on astrocytes.

### Expression Studies

MPTP administration significantly increased the mRNA level of iNOS (*p* < 0.001) (Figure 2C), which was significantly restored in the BME + MPTP (*p* < 0.01) and MPTP + BME (*p* < 0.05) groups.

### Molecular Docking with Different Targeted Ligand-Receptor Proteins

**FIGURE 3:**
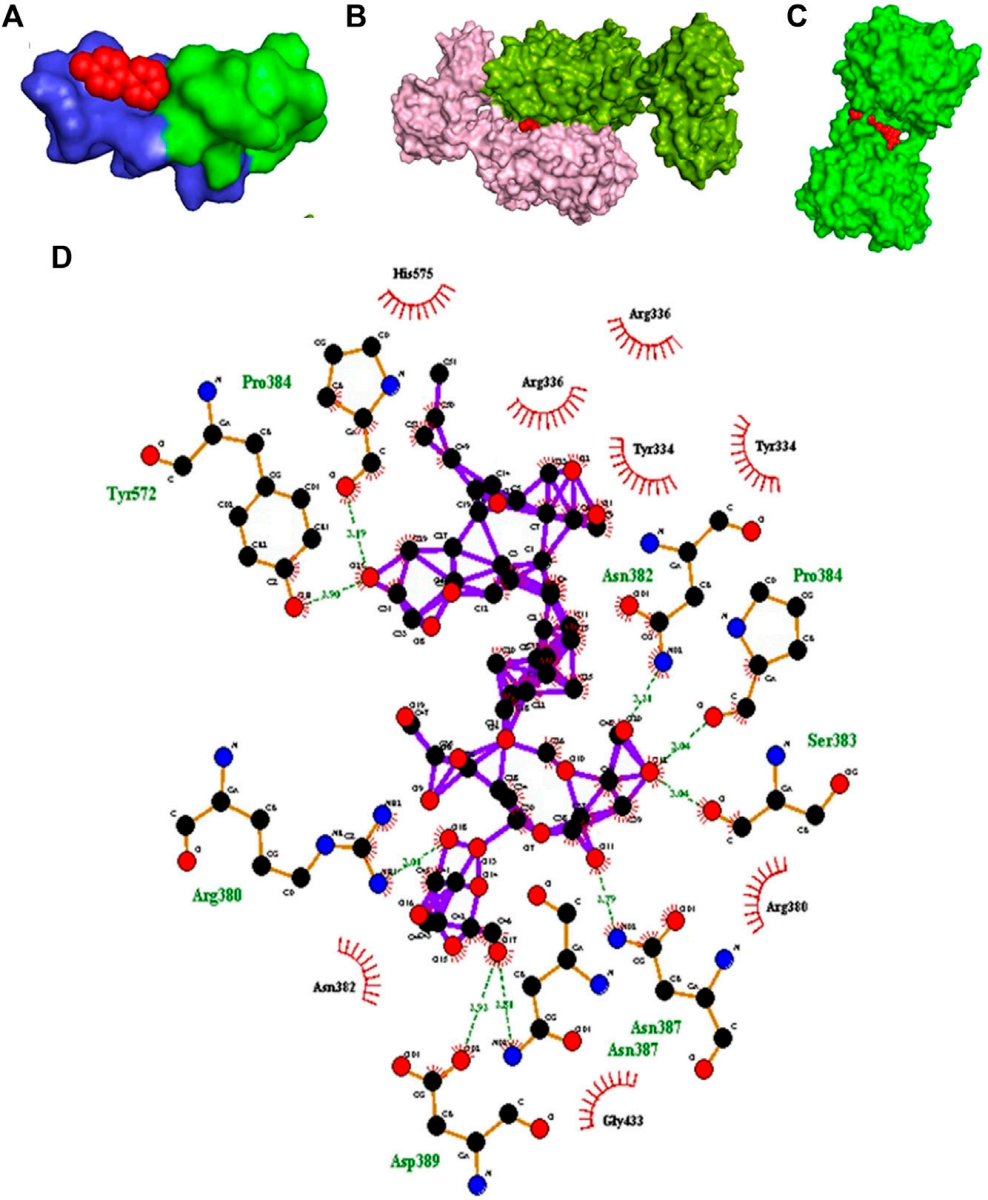
*In silico* study. **(A)** Molecular surface structure of D2 dopamine receptor (PDB: 5AER) (Chain B-light green and Chain C-blue) in complex with MPP+ (red in color). **(B)** Molecular surface structure of D3 dopamine receptor (PDB: 3PBL) (Chain A-light pink and Chain B-green color) in complex with MPP + toxin (red color). **(C)** Molecular surface structure of KEAP1 protein (PDB: 3ZGC) (green color) in complex with bacopaside-XII (red sphere). **(D)** LigPlot depicting schematic representation of hydrogen bonds and hydrophobic interactions between bacopaside-XII and KEAP1 protein.

Molecular docking tools were employed to explore the oxidative stress activity of BM phytochemicals. Docking results of dopamine receptor and ligand MPP^+^ showed very high affinity. MPP^+^ binds with D2 and D3 dopamine receptor with a binding energy of −4.9 and 7.9 kcal/mol, respectively, as shown in molecular surface structure (Figures 3A,B). The docking scores for known standard inhibitors of D2 (eticlopride, raclopride) and D3 (raclopride) dopamine receptor were −4.7, −4.5, and −6.7 kcal/mol, respectively (Table 1).

**TABLE 1:** Binding energy of *B. monnieri* phytoconstituents and standard inhibitors with different proteins involved in antioxidant defense system, locomotion physiology, and dopamine degradation pathway.

S. No	PubChem ID	Ligand name	Proteins*										
			P1	P2	P3	P4	P5	P6	P7	P8	P9	P10	P11
1	5213	Silybin	—	—	—	—	—	—	−10.3	—	—	—	—
2	6407	Chloral	—	—	—	—	—	—	—	—	−3.6	—	—
3	26757	Selegiline	—	−7	−7	−5.1	—	—	—	—	—	—	—
4	57267	Eticlopride	—	—	—	—	—	—	—	—	—	—	−4.7
5	121938	LY134046	—	—	—	—	−7.1	—	—	—	—	—	—
6	400769	CDDO-Me	—	—	—	—	—	—	—	−9.9	—	—	—
7	3033769	Raclopride	—	—	—	—	—	—	—	—	—	−6.7	−4.5
8	5280863	Kaempferol	—	—	—	—	—	−10.4	—	—	—	—	—
9	5464105	Nitecapone	−6.3	—	—	—	—	—	—	—	—	—	—
10	9876264	Bacopaside II	−6.8	−8.2	−9.8	−9	−7.6	−10.4	−9.1	−11	−8.9	—	—
11	10605023	Bacopasaponin G	−7.9	−9.9	−8.8	−9.2	−8.5	−9.4	−8.6	−11	−9	—	—
12	10629555	Bacopaside X	−8	−9.5	−9.8	−9.3	−8.3	−9.9	−9.8	−10.8	−10.2	—	—
13	10865594	Bacopaside IV	−7.4	−9	−10.3	−10	−8.2	−9.2	−9.5	−11.5	−8.9	—	—
14	11079173	Bacopaside A	−5	−6.4	−6.4	−6.3	−5.4	−6.5	−7.7	−7.3	−7	—	—
15	11113741	Bacopaside B	−8.4	−8.6	−8.3	−9.2	−7.7	−6.8	−8.1	−8.5	−9.3	—	—
16	11145924	Bacopaside C	−7.2	−10.1	−9.1	−7.9	−7.3	−7.8	−10.3	−10.1	−9.8	—	—
17	11949626	Bacopaside N1	−7.3	−8.8	−8.8	−8.6	−7.9	−8.7	−9.2	−10.9	−8.6	—	—
18	15922618	Bacopaside III	−7	−10	−10.1	−10.1	−8.9	−9.8	−10.1	−12.6	−9.7	—	—
19	16216038	Bacosaponin F	−7.1	−8.1	−8.7	−8.3	−7.8	−8.8	−7.5	−9.2	−9.6	—	—
20	21574494	Bacopaside N2	−7.4	−10.8	−8.4	−9.3	−7.8	−9.5	−10.1	−10	−9.3	—	—
21	21599442	Bacopaside I	−7.7	−10.6	−9.3	−8.9	−8	−9.6	−9.5	−11.1	−9.2	—	—
22	44421667	Bacopaside VII	−6.9	−10.4	−10.3	−9.6	−8.2	−9.7	−10.2	−10.4	−9.4	—	—
23	44421668	Bacopaside I	−7.2	−10.3	−9.4	−8.9	−7.9	−9.2	−10.2	−10.8	−9.1	—	—
24	53398644	Bacoside A	−6.8	−7.5	−7.4	−7.8	−7.6	−7.7	−8.7	−11.3	−7.5	—	—
25	71312546	Bacopaside I	−7.9	−10.1	−10.1	−9.3	−8	−8.9	−9.7	−10.4	−9.7	—	—
26	90472275	Bacopaside II	−7	−10.3	−9.3	−8.9	−9.1	−8.6	−10.8	−9.9	−9.1	—	—
27	91827005	Bacoside A3	−7	−8.8	−8.7	−8.8	−7.9	−8.7	−9.8	−9.9	−8.8	—	—
28	101062564	Bacopasaponin E	−7	−8.7	−10.7	−9	−8.2	−8.9	−8.8	−11	−9.5	—	—
29	1,01219808	Bacopaside V	−8	−11.1	−11.2	−9.6	−8.2	−9.8	−10	−12	−10.1	—	—
30	101995276	Bacopasaponin A	−8.1	−9.1	−9.7	−9.2	−8.2	−9.2	−9.8	−12.3	−8.9	—	—
31	101996847	Bacopasaponin B	−7.5	−10	−9.4	−10.3	−8.4	−8.8	−10	−10.4	−8.9	—	—
32	101996848	Bacopasaponin C	−8.2	−9.8	−9.5	−9.1	−8.7	−9.9	−9.5	−10.2	−9.1	—	—
33	102000288	Bacopasaponin D	−7.4	−11.8	−11.9	−9.3	−7.7	−8.5	−10	−9.9	−8.7	—	—
34	102080690	Bacopaside VI	−7.7	−9.8	−9.4	−9.1	−9.2	−9.4	−10.4	−11.4	−9.8	—	—
35	102080691	Bacopaside VII	−7.3	−9.7	−9.3	−9.5	−8.6	−10	−10.2	−10.1	−8.6	—	—
36	102080692	Bacopaside VIII	−7.2	−8.4	−9.5	−8.9	−8.4	−9.1	−8.9	−10.3	−8.3	—	—
37	102418532	Bacopaside XI	−8	−9.5	−11.2	−8.9	−8.8	−9.2	−10.2	−10.3	−8.2	—	—
38	102418533	Bacopaside XII	−11.1	−14	−14.8	−13.3	−13.8	−12.9	−13.1	−14.7	−15.5	—	—
39	118856250	Bacopasaponin C	−8.2	−9.8	−10.4	−9.4	−8.5	−9.3	−10	−10.3	−9.6	—	—
40	39484	MPP^+^	—	—	—	—	—	—	—	—	—	−7.9	−4.9

^*^ *Proteins*. P1: COMT; P2: MAO-A Chain A; P3: MAO-A Chain B; P4: MAO-B; P5: PNMT; P6: PST; P7: UGT; P8: KEAP1; P9: ALDH1A1; P10: D3 dopamine receptor; P11: D2 dopamine receptor (S. no. 1 to 9 represent standard ligand for their respective protein).

Major phytoconstituent of BM phytochemicals, bacosides, bacopasides, and bacopasaponins showed interesting binding affinity with KEAP1 receptor protein (Table 1). The docking studies revealed that bacopaside-XII has the highest affinity for KEAP1 and binds with the lowest energy (−14.7 kcal/mol) among thirty docked compounds as shown in molecular surface structure (Figure 3C). Out of 30, 27 compounds showed better binding affinity than the standard KEAP1 inhibitor CDDO-Me (−9.9 kcal/mol). Bacopaside A (PubChCID-11079173), bacopaside B (PubChem CID-11113741), and bacopasaponin F (PubChem CID -16216038) showed comparatively lesser binding affinity than the standard. The docking values for the entire test BM phytoconstituents and standard inhibitor with KEAP1 protein are given in Table 1. Residues Pro384, Tyr572, Asn382, Pro384, Ser383, Arg380, Asn 387, and Asp389 of KEAP1 receptor protein were involved in the formation of hydrogen bonds (nine) with bacopaside-XII (Figure 3D).

**FIGURE 4:**
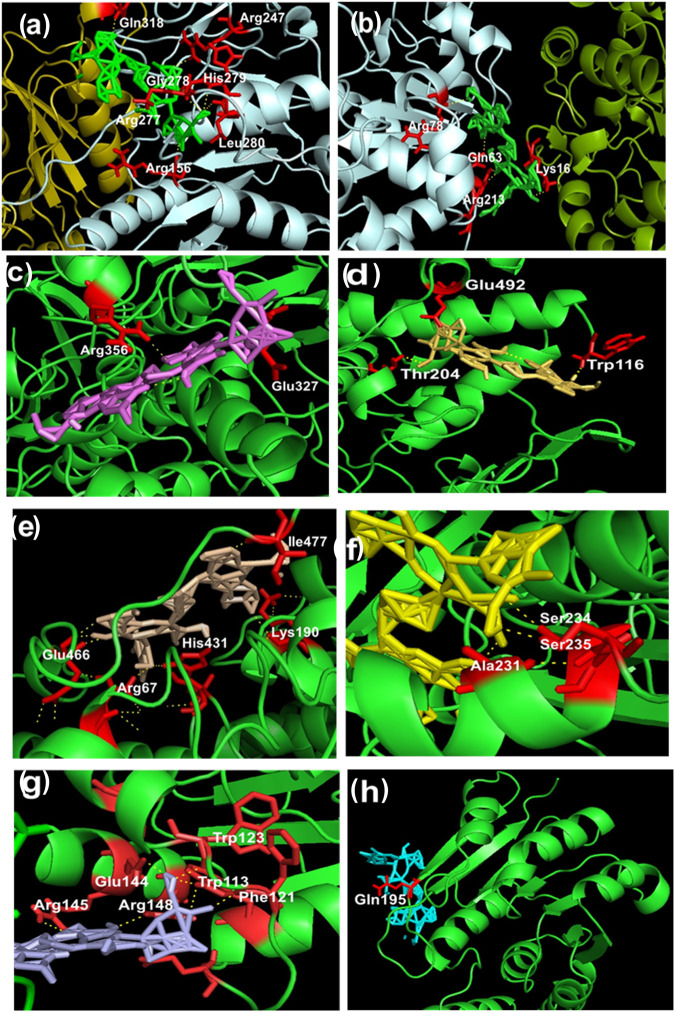
**(A–H)** Representation of intermolecular hydrogen bonds between bacopaside-XII and various dopamine degradation enzymes. **(A)** UGT (PDB: 3PBL): Chain A pale cyan; Chain B deep olive; red color residues and green color bacopaside-XII. **(B)** PST (PDB: 2A3R): Chain A split pea; Chain B pale cyan; red color residues and green color bacopaside-XII. **(C)** MAO-A Chain A (PDB: 2BXS): Chain A green color; red color residues and violet color bacopaside-XII. **(D)** MAO-A Chain B (PDB: 2BXS): Chain B green color; red color residues and yellow orange color bacopaside-XII. **(E)** MAO-B (PDB: 4A79): Chain A and Chain B in green color; red color residues and wheat color bacopaside-XII. **(F)** ALDH1A1 (PDB: 4WP7): Chain A green color; red color residues and yellow color bacopaside-XII. **(G)** PNMT (PDB: 4MQ4): Chain A and Chain B in green color; red color residues and light blue color bacopaside-XII. **(H)** COMT (PDB: 3BWM): Chain A green color; red color residue and cyan color bacopaside-XII.

BM phytochemicals were also docked with the enzymes (MAO-A, MAO-B, COMT, ALDH, PNMT, PST, and UGT) involved in dopamine degradation pathway, and results were compared with the standard known inhibitors (Table 1 and Figures 4A–H).

Out of the entire test BM phytoconstituents, bacopaside-XII showed the highest binding energy of −13.3 kcal/mol with MAO-B enzyme (Table 1). By using the PyMOL visualization tool, the different residues of MAO-B, namely, Ile477, Glu466, Lys190, His431, and Arg67, were identified to be involved in the formation of hydrogen bonds with bacopaside-XII (Figure 4E). Bacopaside-XII binds to COMT, MAO-A Chain A, MAO-A Chain B, MAO-B, PNMT, PST, UGT, and ALDH1A1 with highest binding affinity than other docked test compounds, ranging from −11.1 to −15.5 kcal/mol (Table 1). The PyMOL visualization tool revealed that different residues of UGT (Gln318, Arg247, Gly278, His779, Arg277, Leu280, Arg156), PST (Arg78, Gln63, Lys16, Arg213), MOA-A (Arg356, Gln327, Glu492, Trp116, Thr204), MAO-B (Ile477, Glu466, Lys190, His431, Arg67), ALDH1A1 (Ser234, Ser235, Ala231), PNMT (Trp123, Glu144, Trp113, Arg145, Arg148, Phe121), and COMT (Gln195) were involved in the formation of hydrogen bonding with bacopaside-XII (Figures 4A–H).

## Discussion

An understanding of the detailed mechanism of PD progression is required for the development of an effective neuroprotective therapeutic approach to halt or to slow the disease progression. The mouse model of MPTP-induced PD is widely used to test new treatment interventions for the disease. Intraperitoneal administration of MPTP in mice causes motor-function-related problems, activation of proinflammatory cytokines, oxidative stress, and alterations in the neurotransmitters level (Singh et al., 2016). 1-Methyl-4-phenylpyridinium (MPP+) is a toxic metabolite of MPTP, which specially inhibits complex I (NADH dehydrogenase) of the mitochondrial electron transport chain leading to a decrease in ATP generation, thereby causing death of dopaminergic neurons in the SNpc region of the brain (Hutter-Saunders et al., 2012). Loss of dopaminergic neurons is not only due to decreased mitochondrial function but also due to the increased levels of proinflammatory cytokines and reactive oxygen species (ROS). Degeneration of the membrane lipids produces high levels of intracellular ROS, which ultimately leads to the loss of membrane integrity and dopaminergic neurodegeneration. Oxidative stress is one of the major causes of neurodegeneration. Dopaminergic neurons of the nigrostriatal area are more susceptible to oxidative damage as this area is allied with high-energy consumption and has low levels of the antioxidant GSH (Yamaguchi and Shen, 2007). Therefore, ROS scavenging antioxidants might play a crucial role in preventing PD progression by inhibiting ROS-induced neurodegeneration.

*Bacopa monnieri* (L.) *Wettst* is a well-known dietary antioxidant. Several *in vitro* and animal studies have established that BME inhibits oxidative stress by reducing the formation of free radicals in the brain (Kumar et al., 2012; Shinomol and Bharath, 2012). Studies also showed the neuroprotective role of BME, but there is a lacuna in the studies of neuroprotective and neurorescue effects of BME. To the best of our knowledge, this study is the first to demonstrate neuroprotective and neuroreparative (neurorescue) effect of BME in the MPTP-induced model of PD.

Animals were treated with BME (40 mg/kg bw) orally before and after MPTP administration. The BME dose was chosen based on our earlier studies, where BME has been found to protect against MPTP-induced neurodegeneration (Singh et al., 2016). Although several groups have characterized the chemical components of BME, active components or chemical entities responsible for neuroprotective and neurorescue action of BME are not evidently defined yet.

In the present study, mice treated with MPTP showed a huge decline in motor activity as evaluated by different neurobehavioral tests (grip strength, footprinting, and rotarod test), which is in accordance with previous studies (Singh et al., 2016). The decreased time spent by the MPTP-treated mice on the rotarod is attributed to loss of the dopaminergic neurons within the basal ganglia, especially in the mid brain region of substantia nigra (Schwarting et al., 1991). Previous studies showed that decrease in the stride length is significantly associated with the magnitude of neuronal loss in the SN region of the brain (Fernagut et al., 2002). Oral BME administration before or after MPTP lesioning significantly improved the motor functions, suggesting neuroprotective/neurorescue effect of BME on dopaminergic neurons against MPTP-induced toxicity. Our behavioral studies indicate that BME might play an essential role in enhancing the grip power in the PD mice model, supporting its neuroprotective/neurorescue potential. Our findings correlate well with those of the earlier studies from our group as well as from others (Schwarting et al., 1991; Fernagut et al., 2002; Singh et al., 2016).

Results of our study demonstrated that injection of MPTP toxin upregulated the levels of lipid peroxides and nitrite and reduced the GSH level in the substantia nigra region. Administration of BME (pre- and posttreatment) reduced the oxidative stress. Our results can be explained by the fact that BME possesses strong radical scavenging activity (Simpson et al., 2015; Singh et al., 2016).

Dopamine is the most important neurotransmitter involved in the control of motor activities and movement. Previous studies showed that, in human PD patients, the level of catecholamines is lower compared with healthy controls (Hinterberger, 1971; Piggott et al., 1999). We postulate that an increase in the levels of dopamine in both treatment groups (pre- and posttreatment) might be due to the capability of BME to prevent degradation of DA or inhibit reuptake of DA. It might also be possible that the increased levels of DA in the BME-treated mice might be due to the neuroprotective effect of BME on dopaminergic neurons. Nevertheless, our findings that BME treatment increases the levels of DA corroborate with previous studies (Ghosh et al., 2009).

MPTP intoxication can stimulate the production of various proinflammatory molecules within the substantia nigra region (Ghosh et al., 2009). Our previous study showed that treatment with BME could attenuate the increased expression of these proinflammatory molecules in MPTP-induced mice (Singh et al., 2017). We found that MPTP intoxication led to a marked increase in gliosis as evidenced by increased number of GFAP positive neurons in the SNpc region. Thus, MPTP administration causes microglial activation and increases the expression of iNOS in the substantia nigra region of the brain (Liberatore et al., 1999), leading to the production of nitric oxide, eventually causing neuronal death. Our results showed that MPTP treatment increased the production of nitric oxide possibly by increasing the levels of iNOS. Increased iNOS activity enhances nitric oxide production, which promotes dopaminergic neuronal death by nitrosative/oxidizing damage and other respiratory deficiency (Tsang and Chung, 2009). Our results showed that MPTP intoxication increases the number of GFAP expressing cells and the transcript levels of iNOS in mice, and BME (pre- and posttreatment) ameliorates these changes. Thus, the neuroprotective and neurorescue effects of BME may involve the regulation of antioxidant enzymes and transcription factors by compounds present in BME.

In agreement with previous studies, our study showed that the toxin MPTP induces phenotypes associated with Parkinson’s disease in mice such as decreased levels of dopamine and GSH; increased levels of MDA, iNOS, and TNF-α; and increased numbers of GFAP positive astrocytes. Moreover, our results suggest that pretreatment with BME is more effective in ameliorating the neurotoxicity in the MPTP-induced mice model of PD as compared with posttreatment of BME. This indicates that prior intake of BME might be more helpful in preventing neurodegeneration as well as in slowing down the disease progression (neuroprotective effect) as compared with post intake of BME (neurorescue effects). However, further studies are required to understand the mode of action of BME and to identify the active component(s) of BME responsible for neuroprotective and neurorescue effects.

Our *in silico* study suggests that BM phytoconstituents (mainly bacopaside-XII) have the ability to block KEAP1 protein. Thus, it may be inferred that inhibition of KEAP1 protein further inhibits the Cullin 3-mediated ubiquitination of Nrf2 protein and thereby upregulates the expression (through Nrf2) of antioxidant enzymes (Figures 3E,F). We, therefore, postulate that these phytochemicals act as natural drug to counter KEAP1-mediated oxidative stress.

**FIGURE 5:**
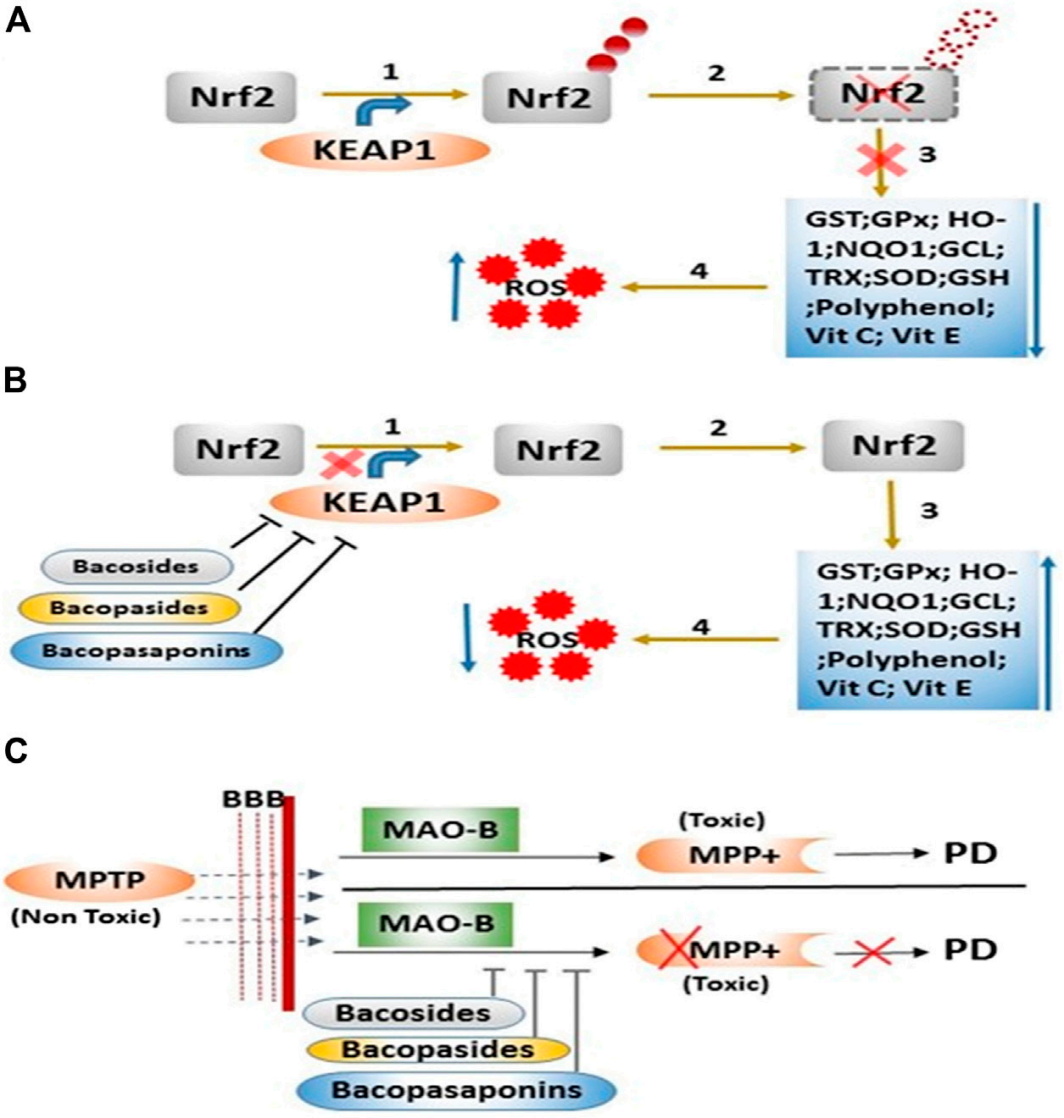
Mode of action of *B. monnieri* phytoconstituents on the regulation of the antioxidant system and metabolic degradation of MPTP. **(A)** Step 1: ubiquitination of Nrf2 protein via KEAP1 protein, Step 2: degradation of Nrf2 protein, Step 3: downregulation of various enzymatic and nonenzymatic antioxidants, and Step 4: enhancement of reactive oxygen species (ROS). **(B)** Step 1: inhibition of KEAP1 protein via *B. monnieri* phytochemicals, Step 2: inhibition of Nrf2 degradation, Step 3: expression of enzymatic and nonenzymatic antioxidants, and Step 4: decrease in the ROS level. **(C)** *B. monnieri* phytochemicals inhibit the degradation of MPTP (nontoxic) into MPP+ (toxic) product through the binding with catalyzing enzyme MAO-B.

The enhanced levels of DA in the MPTP + BME group could be due to the protective effect of BME on dopaminergic neurons. Another possibility is that BM phytoconstituents may inhibit the enzymes involved in the DA degradation pathway. There are several distinct dopamine degradation pathways that act via the set of enzymes such as monoamine oxidase A and B (MAO-A and -B), catechol-O-methyl transferase (COMT), aldehyde dehydrogenase (ALDH), UDP-glucuronosyltransferases (UGT), phenol sulfur-transferase (PST), and phenylethanolamine N-methyltransferase (PNMT) acting in sequence (Meiser et al., 2013). We performed the molecular docking study to dock various enzymes involved in DA degradation, with BM phytoconstituents. Result showed that BM phytochemicals (bacosides, bacopaside, and bacosaponins) have the ability to inhibit all the abovementioned enzymes involved in DA degradation (Figure 5; Table 1).

## Conclusion

Both, *in vivo* and *in silico* data indicate that BM phytochemicals have the ability to maintain DA concentrations in the mice brain by either increasing dopamine synthesis or inhibiting DA degradation. An understanding of the pathophysiology and etiology of PD at cellular and molecular levels is the clinical need of the hour. For neuroprotective disease-modifying therapy, identifying the molecular targets is essential in the field of PD basic research. Thus, our study may offer a therapeutic approach for treating this neurodegenerative disease.

## Data Availability

The original contributions presented in the study are included in the article/Supplementary Material; further inquiries can be directed to the corresponding author.
